# Dietary Patterns and Nutritional Status of Polish Elite Athletes

**DOI:** 10.3390/nu17162685

**Published:** 2025-08-19

**Authors:** Florentyna Tyrała, Barbara Frączek

**Affiliations:** 1 Department of Sports Dietetics, Gdansk University of Physical Education and Sport, Kazimierza Gorskiego 1, 80-336 Gdansk, Poland; 2 Department of Sports Medicine and Human Nutrition, Institute of Biomedical Sciences, University of Physical Culture in Krakow, Jana Pawla II 78, 31-571 Krakow, Poland; barbara.fraczek@awf.krakow.pl

**Keywords:** dietary habits, diet, nutrition, athletic performance, sport, health, food frequency questionnaire

## Abstract

Background: Rational dietary patterns and adequate nutritional status support athlete health and performance, while unhealthy habits may impair these outcomes. This study aimed to identify dietary patterns among Polish professional athletes using a food frequency questionnaire and assess their correlations with nutritional status indicators. Methods: Participants included 226 elite Polish athletes (aged 16–39 years; 87 women, 139 men) from various sports disciplines. Dietary intake was assessed using a food frequency questionnaire, and dietary patterns were identified through principal component factor analysis. Nutritional status was evaluated using anthropometry, bioelectrical impedance, and selected blood biochemical markers. Spearman’s rho correlations were applied to explore associations between dietary patterns and nutritional status. Results: Eight dietary patterns were identified: ‘High-fat’, ‘Sweets and beverages’, ‘Potentially rational’, ‘Vegetables and fruits’, ‘Meat and flour’, ‘Low-fat’, ‘Dairy’, and ‘Juices’. Of the two patterns considered unhealthy, ‘High-fat’ was associated with anthropometric indices—positively with the slenderness index and negatively with body mass index, particularly among men. Positive correlations with favorable nutritional indicators were observed for the ‘Vegetables and fruits’ pattern (arm muscle circumference, BMI, serum uric acid, hydration status), ‘Low-fat’ (body fat percentage), ‘Dairy’ (serum creatinine), and ‘Juices’ (serum creatinine, total protein, chlorine, uric acid). Conclusions: Our findings suggest that the identified dietary patterns are original and specific to Polish professional athletes. Determining the relationships between nutritional factors and anthropometric and biochemical indices may inform dietary modifications among athletes to ensure optimal nutritional status.

## 1. Introduction

Nutritional practices have a major impact on the exercise capacity of athletes and general health [1,2], and proper nutrition is an essential factor for optimizing athletic performance and adaptation [3]. With an optimal diet, it is possible to achieve full, genetically programmed mental and physical development, whilst maintaining the body’s immunity system [4]. Interpretation of athlete nutritional, medical, anthropometric (anthropometric measurements and anthropometric indicator), and biochemical (peripheral blood parameters, biochemical markers of peripheral blood, and general urine analysis) data ensures that nutritional intake can be objectively assessed. For example, the measurement of food intake, diet, and nutritional status—closely interrelated components—has a significant impact on health status [5] and, arguably, on athletic potential. Adherence to the principles of proper nutrition is a key determinant of growth, development, and athletic performance. Physical training substantially increases energy expenditure, thereby elevating the demand for energy, water, macronutrients (proteins, carbohydrates, fats), vitamins, minerals, and other bioactive compounds. Nutritional support must be tailored not only to these elevated needs but also to individual training variables, such as exercise intensity, type, and duration, as well as the timing of nutrient intake in relation to physical activity. An athlete’s diet should consist of appropriately varied, balanced meals based on natural food sources, providing all essential nutrients. Optimal nutritional status is achieved when dietary intake aligns with established dietary reference values, which vary depending on age, sex, body weight, physiological status, and physical activity level. Moreover, the effectiveness of dietary strategies depends on their integration with correct physical activity practices, personalized energy periodization, and meal timing—factors that play a crucial role in supporting performance, recovery, and adaptation [1,6]. Conversely, inadequate nutrient or fluid intake can impair strength, endurance, concentration, and post-exercise recovery, while increasing the risk of injury. Both nutrient deficiencies and excesses may lead to adverse effects at cellular, tissue, and systemic levels, with symptom severity depending on the type and duration of the nutritional imbalance [4].

Recent international research has increasingly used validated dietary assessment tools such as the Athlete Diet Index (ADI) or diet quality indices to evaluate diet quality in elite athlete populations. For example, a scoping review found that many adult sports cohorts worldwide exhibit poor intake of whole grains, fruits, and dairy, despite adequate protein intake [7]. Similar observations were reported in a large cohort of elite Australian athletes assessed via the ADI, where diet quality varied significantly across disciplines and between sexes [8]. Moreover, a longitudinal study of elite female basketball and volleyball players demonstrated that although athletes frequently did not meet energy and macronutrient recommendations, body composition and performance outcomes often improved, suggesting complex adaptation mechanisms [9]. However, despite these insights, there is a clear lack of data regarding dietary patterns and their biochemical associations among elite Polish athletes—a gap our study directly addresses.

Dietary patterns [DPs] represent a group of multiple characteristics or commonly occurring traits that describe human nutrition [10]. Identification of athlete DPs ensures that the relationship between a characteristic dietary model and other factors (e.g., health status, body composition parameters, nutrient supply, type of diet, and degree of risk of deficiencies) can be determined, as a starting point to improve these outcomes [11]. Dietary patterns have been characterized in various population groups [8,12,13,14,15,16,17,18]; however, when it comes to publications concerning physically active individuals, their number is limited.

Crucially, the use of nomenclature in these studies, such as ‘dietary pattern/s,’ ‘eating pattern/s,’ ‘dietary profile/s,’ and ‘food pattern/s’, often did not involve identification of DPs using statistical techniques, but rather was a colloquial term for a way of eating or type of diet. A number of studies have evaluated dietary behaviors in athletic populations using tools such as food frequency questionnaires (FFQs), food diaries, or structured interviews [8,16,18,19,20,21,22,23,24,25,26,27,28,29,30,31,32]. In these studies, ‘patterns’ referred to observed consumption habits—e.g., macronutrient intake, hydration, or food group frequency—rather than statistically defined constructs. Among the reviewed literature, only Frączek (2013) [17] and Kopiczko et al. (2025) [33] employed statistical methods to identify dietary patterns (DPs) based on interrelationships within food frequency data, utilizing principal component analysis (PCA) and cluster analysis, respectively. Specifically, Frączek (2013) delineated several distinct dietary patterns characterized by particular food group loadings through PCA [17], thereby elucidating meaningful dietary groupings within the study population. Similarly, Kopiczko et al. (2025) applied cluster analysis to categorize participants into discrete dietary clusters, uncovering associations between these clusters and relevant health outcomes [33]. This distinction underscores that only a limited number of studies applied rigorous statistical techniques for the identification of DPs, whereas the remaining literature predominantly referred to dietary habits in a more descriptive or colloquial manner without formal pattern derivation. According to our knowledge, studies evaluating the relationship between DPs and nutritional status in professional or elite Polish athletes has not yet been performed. Determining the relationships between nutritional factors and anthropometric and biochemical indices may help in formulating a structured plan to create a rational or optimal diet for athletes, thereby supporting athletic training to achieve a higher level of physical performance, recovery, and adaptation, as well as better health outcomes. This detail plays an important role, both theoretically and practically, for athletes, their coaches, and their nutrition specialists.

In this study, we aimed to extract DPs based on the frequency of food consumption of Polish professional athletes, and to identify the correlation between DPs and nutritional status of Polish professional athletes. The specific objectives included assessing the frequency of consumption of food products, dishes, and beverages; analyzing somatic characteristics, anthropometric indicators, and blood and urine test results; and evaluating the relationships between dietary profiles and nutritional status with consideration of both female and male athletes. The present study was guided by the following research questions:

1. What are the nutritional status indicators observed in the studied athletes?
2. Which dietary patterns are characteristic of Polish athletes?
3. Do the identified dietary patterns correlate with nutritional status indicators?
4. Do dietary patterns and nutritional status differ according to sex?

Based on these research questions, the following hypotheses were formulated:

1. The identified dietary patterns (DPs) among Polish athletes exhibit similarities to those previously described in the literature.
2. Non-healthy dietary patterns are positively associated with higher anthropometric indicators (BMI, WHR, AMC, %FM) and negatively associated with the slenderness index.
3. Non-healthy dietary patterns occur more frequently in men than in women.
4. Prudent dietary patterns are positively correlated with indicators of proper nutritional status.

## 2. Materials and Methods

### 2.1. Study Population and Study Design

We conducted this study in 2017–2019 on a cohort of 226 (87 W and 139 M) professional Polish athletes, aged 16–39 years (mean age was 22.8 ± 5.48), representing various sport disciplines (74.8%, *n* = 169, individual sports and 25.2%, *n* = 57, team-based sports). The respondents were dominated by athletics (31.0%, *n* = 70), volleyball (12.8%, *n* = 13) and cross-country skiing (8.0%, *n* = 18). The main inclusion criteria were as follows: practicing competitive sports for at least 3 years; undertaking regular physical effort at least 5 times a week across more than 1 h training units; and participation in national and/or international competitions. The exclusion criteria were as follows: age below 16 or above 39 years; smoking; chronic alcohol consumption; recent surgeries or illnesses affecting the cardiovascular system, musculoskeletal system, liver, kidneys, or metabolic functions; and the use of anti-inflammatory drugs or antibiotics during the study period. The study participants were recruited using purposive sampling through collaboration with national sports federations, regional athletic associations, and professional sports clubs across Poland. Coaches and team medical staff were contacted to assist in identifying eligible athletes based on the inclusion and exclusion criteria. Athletes were then invited to participate voluntarily and provided written informed consent. A total of 250 athletes were initially approached, of whom 226 met all criteria and agreed to take part in the study. All participants were assessed during the preparatory period preceding the competition season, which helped minimize the potential influence of seasonal variation on the analyzed indicators. This research was approved by the Bioethics Committee at the Regional Medical Chamber in Krakow on 14 March 2017, number 37/KBL/OIL/2017.

### 2.2. Dietary Assessment

To assess the dietary habits of participants, we implemented a food frequency questionnaire (FFQ) previously validated in the Department of Sports Medicine and Human Nutrition, University School of Physical Education, Krakow [34]. The participants independently completed the survey questionnaire in paper form, in the presence of the interviewer. The FFQ was structured to reflect food habits of a Polish population and consisted of 33 groups of food products, dishes, and drinks from 13 food groups: unsweetened drinks; vegetables and vegetable preserves (including vegetable juices); fruit and fruit preserves (including fruit juices); cereal products, potatoes, flour and potato dishes, and legumes; milk and dairy products; eggs (including egg dishes); meat and meat products; fish and fish preserves; vegetable fats; animal fats; sugar and confectionery; other drinks (sweetened, energizing, alcohol); and fast food. Athletes were asked how often they consumed each item per day, per week, and per month during the last year. The FFQ uses a six-point scale of consumption, as follows: never (1), 1–3 times a month (2), once a week (3), several times a week (4), once a day (5), and several times a day (6). To interpret the average reported values of the FFQ, the following rankings were used: never (1.49–1.00), 1–3 times a month (1.50–2.49), once a week (2.50–3.49), several times a week (3.50–4.49), once a day (4.50–5.49), and several times a day (5.50–6.00).

### 2.3. Anthropometric Assessment and Blood and Urine Biomarkers

We assessed athletes using the following anthropometric measurements: body height (cm), body weight (kg), midarm circumference at rest (MAC, cm), waist circumference (cm), hip circumference (cm), skinfold (biceps skinfold, triceps skinfold, subscapular skinfold, and suprailiac skinfold) thickness (mm), and bioelectrical impedance: total body water analysis (%). To minimize variations in anthropometric measurements, all data were collected by the same experienced staff member, i.e., a trained professional with expertise in anthropometric measurement techniques. All body measurements we performed on the right side of body, and the final result was the average of three assessments [35]. To assess body fat distribution, we used the waist–hip ratio (WHR) expressed as the ratio of waist circumference to hip circumference [10]. We used MAC to derive arm muscle circumference (AMC) (AMC = MAC − 3.14 × triceps skinfold thickness) [36], as an indicator of protein stores. A body mass index (BMI, weight in kg divided by height in m^2^) was also calculated [37]. To evaluate athlete body type, we used a slenderness index that was calculated by dividing height by the cubic root of weight [38]. We determined fat mass percentage (%FM) from the Siri formula [39] using linear regression equations developed by Durnin and Womersley [40].

We measured the following blood biomarkers: peripheral blood count, hemoglobin (HGB), hematocrit (HCT), red blood cells (RBCs), mean corpuscular hemoglobin (MCH), mean corpuscular volume (MCV), mean corpuscular hemoglobin concentration (MCHC), red blood cell distribution width (RDW-CV), platelet count (PLT), and white blood cells (WBCs). Other measures of white blood cell system include, neutrophils (NEUT), lymphocytes (LYMPH), monocytes (MONO), eosinophils (EOS), and basophils (BASO). Biochemical tests of peripheral blood include serum sodium, potassium, chloride, total serum calcium, inorganic phosphorus, serum magnesium, iron, ferritin, serum vitamin B12, total serum protein, serum albumin, serum urea, creatinine, uric acid, glucose in blood, triglycerides level (TG), level of total cholesterol (TC), LDL cholesterol (LDL-c), HDL cholesterol (HDL-c), total serum bilirubin, aspartate transaminase (AST), alanine aminotransferase (ALT), and γ-glutamyl transferase (GGTP). Urine was collected and measured for color, pH, specific gravity (SG), protein, glucose, ketone bodies, bilirubin, urobilinogen, and nitrites.

Due to the fact that blood and urine measurements originated from different diagnostic laboratories, with tests sometimes undertaken using different platforms or methodologies, and some parameters expressed in different units of measurement, we decided to present this data in the form of ranks (1—below norm; 2—within norm; 3—above norm) for analysis. This approach was chosen to ensure comparability across datasets and to minimize potential biases introduced by methodological heterogeneity. Ranking allowed us to harmonize the data despite differences in assay standards and reference ranges, which would otherwise make direct numerical comparison unreliable or misleading. While we acknowledge that the transformation of continuous biochemical values into ordinal ranks may reduce statistical sensitivity and limit the ability to detect subtle variations, we judged this trade-off acceptable in the context of our study, which prioritized consistency and interpretability across heterogeneous data sources. Moreover, categorizing values relative to clinical norms (i.e., below, within, or above reference range) retains essential diagnostic relevance while reducing the influence of laboratory-specific variability.

### 2.4. Derivation of Dietary Patterns

**Figure 1:**
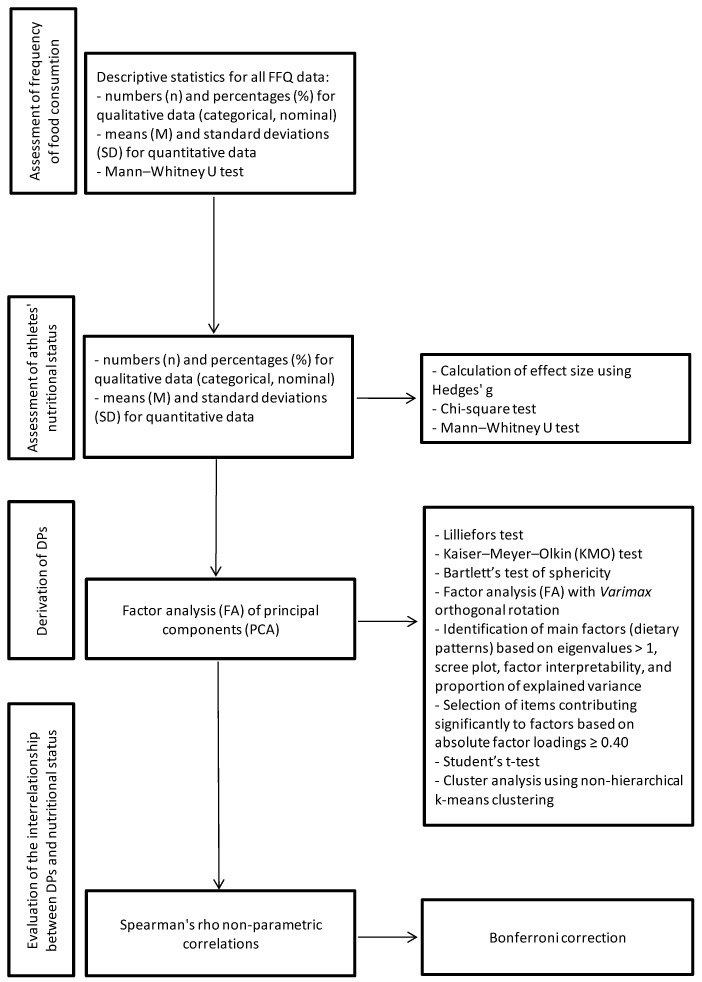
Flow chart of the statistical procedures used in the study.

We empirically derived athlete DPs on the basis of factor analysis (FA) of principal components (PCA) (Figure 1) [41]. The frequency of consumption of the 33 groups of food products, dishes, and drinks constituted the output factors of the variable base. To assess the normality of the factor scores distribution, we used the Lilliefors test. Where appropriate, the Kaiser–Meyer–Olkin (KMO) test (to verify method applicability) and Bartlett’s test of sphericity (to test the null hypothesis of no relationship between the variables) were carried out. The KMO value was 0.69 and the significance of Bartlett’s sphericity was below 0.001, indicating that FA was appropriate to use. *Varimax* orthogonal rotation was performed to maintain independent factors while improving interpretability. We identified main factors (i.e., DPs) according to the eigenvalue (>1), a scree plot, factor interpretability, and the proportion of variance explained by each factor [42]. Items with absolute factor loadings of ≥0.40 were considered to significantly contribute to a given DP. We abandoned interpretation of the remaining factors because they were made up of individual products. The higher the values of the factor loadings, the stronger was the association between a participant’s diet and the DP. We named DPs according to the magnitude of factor scores, published research, and interpretation of overall diet.

### 2.5. Statistical Analyses

We performed statistical analyses using IBM SPSS 21^®^ software (IBM Corp., Amonk, NY, USA). Descriptive statistics were computed for all FFQ questions (Figure 1). We presented data as numbers (*n*) and percentages (%) for qualitative data (categorical, nominal), whereas quantitative data were presented as means (M) and standard deviations (SDs). The chi-square test we used to analyze the relationship between categorical variables and Mann–Whitney U tests were performed to examine differences between two groups for continuous variables. Due to the violation of the assumption of normality, the non-parametric Mann–Whitney U test was employed to examine gender differences. We calculated effect size statistics for two independent samples based on their means, sample sizes, and standard deviations. Since the sample sizes were different, we used Hedges’ g, which provides an alternative effect size measure weighted according to the relative size of each sample. Assuming that the measurements follow a normal distribution and the variances in the samples are not significantly different, we used a Student’s *t*-test to represent intergroup gender differences in each DP [43]. Due to the violation of the normality assumption by many variables and/or the ordinal nature of the measurement, Spearman’s rho non-parametric correlations were used to indicate covariation between nutritional status and the DPs. The strength of these correlations were classified as follows: |r| = 0—no correlation; 0.0 < |r| ≤ 0.1—faint correlation; 0.1 < |r| ≤ 0.3—weak correlation; 0.3 < |r| ≤ 0.5—moderate correlation; 0.5 < |r| ≤ 0.7—high correlation; 0.7 < |r| ≤ 0.9—very high correlation; 0.9 < |r| < 1.0—almost full correlation; and |r| = 1—full correlation [41]. We used the Bonferroni correction to reduce the chances of obtaining false-positive results (type I errors). The corrected alpha level was approximately 0.000133 [43,44].

## 3. Results

### 3.1. Nutritional Status

We presented the anthropometric and nutritional status results in Table 1. Male athletes were significantly (*p* < 0.001) taller (*g* = 1.47) and heavier (*g* = 1.31), and had a lower %FM (*g* = 1.81) than female athletes. In contrast, women had lower values for arm circumference at rest (*g* = 0.77), waist circumference (*g* = 1.28), BMI (*g* = 0.67), AMC (*g* = 1.39), and WHR (*g* = 1.81) compared to their male counterparts (*p* < 0.001). Detailed data on the distribution of the studied athletes (%), by BMI, WHR, slenderness index, AMC, and %FM classification, are provided in the Supplementary Materials: see Table S1.

Generally, most of the athletes (64.0–98.8%, *n* = 144–223) tested had normal blood counts, considering the reference values. Protein–cell abnormalities associated with decreased levels of neutrophils and basophils affected 20.4% (*n* = 17) and 9.3% (*n* = 8) of women, and 12.6% (*n* = 17) and 14.5% (*n* = 20) of men, respectively. This indicates normative biochemical values in most athletes, with the exception of slightly lower percentages for serum calcium (normative value: 86.6%, *n* = 196), inorganic phosphorus (87.4%, *n* = 198), iron (87.1%, *n* = 197), creatinine (83.8%, *n* = 189), vitamin B12 level (85.4%, *n* = 193), albumin (73.9%, *n* = 167), urea (89.5%, *n* = 226), total cholesterol level (74.7%, *n* = 169), LDL-c level (87.9%, *n* = 198), total bilirubin (87.4%, *n* = 197), and AST (82.7%, *n* = 186) (Table 2). We found that men were significantly more likely than women to have higher concentrations of albumin (*p* = 0.014), urea (*p* = 0.002), blood glucose (*p* = 0.002), total bilirubin (*p* = 0.034), and LDL-c (*p* = 0.019), but lower AST values (*p* = 0.015). The majority (89.4%, *n* = 202) of athletes, including 92.5% (*n* = 81) of women and 87.9% (*n* = 122) of men, were deemed to be properly hydrated.

### 3.2. Frequency of Food Consumtion

Of all the foods analyzed, water, sweetened hot drinks, fruits, and vegetables were the most frequently (once a day or more) consumed by athletes (Table S2). Women were more likely than men to consume water (*p* = 0.016), fruits (*p* = 0.001), vegetables (*p* < 0.001), and sweetened hot drinks (*p* = 0.028). The athletes consumed light bread (e.g., wheat, rye, toast, rolls, croissants) daily. On average, several times a week, athletes included white meat, white rice, pasta, small cereals, cold cuts, sausages, wieners, milk, eggs, butter, buckwheat groats, cereals, whole wheat pasta, sweets, fermented dairy drinks, wholemeal bread, cheese, processed cheese, moldy cheese, fried foods, potatoes, and fruit juices. Men consumed light bread (*p* = 0.005); cold cuts, wieners, and sausages (*p* < 0.001); and white rice (*p* < 0.001) more often than women, but their consumption of sweets was lower (*p* = 0.043). Less frequently (~once a week), athletes consumed cottage cheese, red meat, fish, vegetable juices, legume seeds, and sweetened beverages. Women were more likely to eat cottage cheese (*p* = 0.049), but less likely to eat yellow cheese (*p* = 0.018), red meat (*p* < 0.001), and sweetened carbonated and non-carbonated beverages (*p* < 0.001) than men. Occasionally (1–3 times a month), athletes consumed canned, marinated, or pickled vegetables; fast food; oils; margarines and mixes; alcoholic beverages; energy drinks; canned meats; lard; and powdered or ready-made soups. Female athletes were less likely (*p* < 0.001) to choose lard and energy drinks (*p* = 0.008), while men were more likely (*p* = 0.041) to choose vegetable fat sources and alcoholic beverages (*p* = 0.015).

### 3.3. Dietary Pattern Characterization

We identified and labeled eight distinct DPs based on the principal component analysis of FFQ data. These eight factors jointly explained 48.5% of the total variance. Food items with absolute factor loadings ≥0.40 were considered representative of each DP. The rotated factor loadings are contained in Table 3.

The arrangement of variables comprising the designated dimensions, or factor loadings explained as product-factor interdependencies, is presented in Table 3. After analyzing factor content, it was decided to give them appropriate names: pattern 1—‘High-fat’, pattern 2—‘Sweets and beverages’, pattern 3—‘Potentially rational’, pattern 4—‘Vegetables and fruits’, pattern 5—‘Meat and flour’, pattern 6—‘Low-fat’, pattern 7—‘Dairy’ and pattern 8—‘Juices’. These patterns explained 8.70%, 6.79%, 6.17%, 5.73%, 5.65%, 5.44%, 5.31%, and 4.71% of the total variance. Pattern 1: ‘High-fat’ was characterized by light bread, i.e., wheat, rye, toast, rolls, and croissants, which were consumed on average once a day, and potatoes, cold cuts, wieners, sausages, cheese, processed cheese, moldy cheese, and butter, consumed several times a week on average. Pattern 2: ‘Sweets and beverages’ was formed by sweets, consumed on average several times a week, sweetened carbonated and non-carbonated drinks, drunk once a week, alcoholic beverages and energy drinks, consumed 1–3 times a month, and powdered or ready-made soups, which were generally never consumed. Pattern 3: ‘Potentially rational’ included clustered grain products, i.e., white rice, pasta, buckwheat groats, flakes, whole grain pasta, fine groats, and eggs, consumed on average several times a week, legume seeds, consumed once a week, and canned, marinated, or pickled vegetables, consumed 1–3 times a month. Pattern 4: ‘Vegetables and fruits’ was classified by vegetables and fruits, consumed on average once a day. Pattern 5: ‘Meat and flour’ consisted of white meat and fried foods, and was included in the diet on average several times a week. Pattern 6: ‘Low-fat’ grouped red meat, consumed on average several times a week, fish, included in the diet once a week, fast food, consumed 1–3 times a month, and lard, never consumed. Pattern 7: ‘Dairy’ contained milk and fermented dairy drinks, consumed on average several times a week, and cottage cheese, included once a week. Pattern 8: ‘Juices’ was characterized by fruit juices, drunk on average several times a week; and vegetable juices and vegetable and fruit juices, included once a week. Analyzing the differences in DPs, including gender, revealed that the intensity of ‘High-fat’ (*p* = 0.001), ‘Meat and flour’ (*p* = 0.014), and ‘Low-fat’ (*p* < 0.001) DPs was significantly higher in men compared to women (Table S3). Conversely, the ‘Vegetables and fruits’ (*p* = 0.001) and ‘Dairy’ (*p* = 0.033) DPs were more frequent among women than men. No significant intergroup differences were found for the other DPs.

### 3.4. Dietary Patterns and Nutritional Status of Athletes

We showed in Table 4 characteristics of the derived DPs in a select group of athletes.

The ‘High-fat’ DP was weakly, positively correlated with slenderness index, HCT, RBC, iron, urea concentration, TG, and SG, and weakly, negatively associated with BMI, uric acid, and AST (Table 5). Also, a weak relationship was observed for the ‘Sweets and beverages’ DP and PLT, WBC, NEUT, albumin, uric acid, and GGTP. This DP was weakly, but negatively, correlated with AST. The ‘Potentially rational’ DP was weakly and positively associated with BMI and BASO, and weakly, negatively correlated with slenderness index, serum chloride, and ketone bodies. The ‘Vegetables and fruits’ DP was weakly, negatively correlated with BMI, AMC, HGB, HCT, RBC, calcium, uric acid, and SG. A weak, positive correlation was found for the ‘Meat and flour’ DP and HGB, RBC, LYMPH, MONO, ferritin, uric acid, and SG. A negative relationship was observed for %FM and the ‘Meat and flour’ DP. The ‘Low-fat’ DP was weakly and positively correlated with BMI, WHR, HCT, RBC, inorganic phosphorus, ferritin, vit. B12, ALT, GGTP, and SG, and averagely, positively related with urea. A weak, negative association was observed for the ‘Low-fat’ DP and %FM and total serum protein. A weak, negative relationship was found between the ‘Diary’ DP and creatinine. The ‘Juices’ DP was weakly, positively correlated with magnesium, iron, and urea, and weakly, negatively associated with BMI, chlorine, total serum protein, creatinine, and uric acid.

## 4. Discussion

### 4.1. Dietary Patterns and Nutritional Status of Polish Athletes

Based on the comprehensive assessment of dietary intake of elite Polish athletes, we subsequently identified eight DPs. In each DP, the factor loadings were positive, so that an increase in the frequency of consumption of one product or nutrient forming a pattern was accompanied by an increase in the frequency of consumption of other products or nutrients from that profile.

The ‘High-fat’ DP, which was more characteristic of men, was represented by a high consumption of light bread, potatoes, cold cuts, sausages, wieners, and butter. In this study, we showed that the ‘High-fat’ DP was weakly, positively correlated with selected indicators of nutritional status. Indeed, athletes were more likely to consume foods in the discussed DP, and also had higher values of the slenderness index. Accompanying the DP, an increase in HCT, RBC, serum uric acid, and SG values may be indicative of incorrect hydration. Normal SG is in the range of 1.010–1.030 and indicates the kidneys’ ability to concentrate urine, whilst an increase in SG indicates dehydration [45]. Elevated triglyceride levels also correlated, positively, with more frequent consumption of foods specific to the ’High-fat’ DP. An inverse relationship was observed for BMI, serum uric acid, and AST levels, which means that subjects representing this DP were characterized by normal, lower values of BMI and the mentioned biochemical indicators. Laboratory testing confirmed normal AST levels in 83% (*n* = 186) and uric acid levels in 91% (*n* = 205) of the studied athletes. For the majority of athletes (84%, *n* = 189), the BMI values were within a normal range, with men presenting a higher BMI than women. The ‘High-fat’ DP may not negatively affect nutritional status due to the increased energy and fat requirements of professional athletes.

Foods such as refined cereals, processed meats, and potatoes, defining the ‘High-fat’ DP, are repeatedly mentioned in patterns identified by other researchers. Most often, the relationship between a ‘Western’, unhealthy DP and a higher risk of metabolic syndrome is highlighted, which may be related to an inadequate supply of health-positive nutrients. The importance of higher amounts of pro-inflammatory SFAs (saturated fatty acids), which are found in large quantities of foods considered to be unhealthy, has been described by some [46,47]. Contrary to our results, in Western countries, DPs high in fat, sweets, and energy density correlate with a higher BMI [48].

The ‘Sweets and beverages’ DP was formed by sweets, which were consumed several times a week, and sweetened carbonated and non-carbonated, alcoholic, and energy drinks, as well as powdered or ready-made soups, consumed relatively rarely. Increased consumption of bread and sweetened soft drinks was noted among athletes training in modern pentathlon, which was associated with insufficient intake of calcium, fruits, and vegetables [49]. In our sample, athletes avoiding carbonated and non-carbonated sweetened beverages, energy drinks, and alcoholic beverages at the same time sought to consume sweets several times a week, particularly among women. Moderate consumption of sweets was declared by the vast majority of professional athletes (75%) by Frączek (2013) [17], while a daily supply of sweets was reported among 60.9% of rowers [50]. The ‘Sweets and beverages’ DP correlated weakly, but positively, with assessed blood count parameters (PLT, WBC, NEUT) and biochemical indicators (serum albumin, serum uric acid, GGTP). Most athletes had normal blood test results; nevertheless, those presenting a DP characterized by a high intake of sweets, fairly frequent drinking of sweetened beverages, and occasional energy and alcoholic beverages had higher levels of PLT, WBC, and NEUT, as well as the aforementioned biochemical indicators, than reference data. The ‘Sweets and beverages’ DP was also weakly, positively associated with albumin concentration—elevated values of it were shown in 26.1% (*n* = 59) of athletes. Men were significantly more likely than women to have a higher albumin concentration, showing possible dehydration. In contrast, the ‘Sweets and beverages’ DP was inversely related to AST, meaning that athletes presenting this DP had mostly (83%, *n* = 186) correct values for this indicator. In other studies, DPs defined by sweets and/or sugary drinks emerged from large studies on 459 healthy American men and women [13], the Greek population [51], 23,423 Norwegian pregnant women [52], 1231 adults aged 20 years and older (mean age 49.3 years) [53], 1852 military men (20–59 years) [54], 10,089 Korean adults (>19 years) [55], 935 women in Cyprus with 817 in a control group [56], and 610 professional athletes [17].

The ‘Potentially rational’ DP, considered healthier for athletes, included eggs and cereal products (white rice, pasta and small groats, buckwheat groats, flakes, and whole-grain pasta), characterized by high frequency of consumption, and pulses, consumed less frequently, on average once a week. Similarly, in a study on Iranian soccer players, the consumption of grain products was highest for bread (82%, 4–7 times/week), followed by rice (39.9%), pasta (38.5%), and potatoes (27.6%) [21]. In contrast, up to 95% of Brazilians training for pentathlon declared a supply of legumes five or more times a week [49]. Insufficient consumption of grain products has been observed in Greek swimmers and members of the water soccer team [57], athletes performing ultra-endurance exercise [58], Belgian sprinters [59], junior soccer trainers [60], martial arts athletes [61], and representatives of individual sports [62]. Athletes presenting this ‘Potentially rational’ DP achieved a higher BMI. Nevertheless, this may be directly related to lower body fat and higher muscle mass. Research on 3839 Tehran football players found that skeletal muscle mass was significantly related to the consumption of protein-rich foods [21]. A weak, inversely proportional relationship was observed for the slenderness index, ketone bodies in urine, and serum chloride. Individuals who frequently consumed food products classified under the ‘Potentially rational’ dietary pattern exhibited a mesomorph body type, which was significantly more common among women than men (70%, *n* = 61 vs. 44%, *n* = 61). In these individuals, no ketone bodies were detected in urine samples. Over the years, authors have described ‘Rational’ DPs [63,64,65,66] that included similar types of foods to those that compose this DP. Increased consumption of cereal products was associated with a lower risk of obesity among Iranian women [67,68], a finding confirmed in a meta-analysis [69]. Healthier dietary patterns were generally positively associated with healthier lifestyles and a lower BMI [70].

The ‘Vegetables and fruits’ DP included vegetables and fruits, which were consumed at a similar high frequency, i.e., several times a day on average, but more so among women than men. Similarly, the diets of other athletes showed that a significant proportion (65.0%) consumed fruit daily or almost daily [17,60], while the remaining athletes included fruit in their menus two to three times a day [17]. Vegetables and fruits, which serve as medium- and low-glycemic sources of digestible carbohydrates, fiber, minerals, and vitamins (including antioxidants), should be consumed in quantities of at least three and two servings per day, respectively [71,72]. Sufficient fruit and vegetable intake was also found in about 55% of the marathon runners [73]. Insufficient intake of fruits and vegetables, relative to recommendations, was observed among 346 (214 M and 132 W) representatives of individual sports [62]; 62 male professional martial arts athletes [61]; 58 Greek swimmers and members of the water soccer team [57]; athletes performing ultra-endurance exercise [58]; 31 young Belgian sprinters and 29 female sprinters [59]; Uzbekistan athletes [74]; 22 Spanish athletes training in taekwondo, judo, and boxing [75]; 56 training pentathlon [49]; 187 Canadian athletes between the ages of 11 and 18 [76]; and elite kayakers [77]. Vegetable consumption, with a frequency of several times per day, was observed among 100 athletes, including 48% of athletes training professionally and 41% of amateurs [78].

We found a weak, inverse relationship between the ‘Vegetables and fruits’ DP and BMI and AMC, such that achieving a lower, normal BMI and AMC was associated with a higher frequency of fruit and vegetable intake. Considering that the majority of athletes were characterized by normal HGB, HCT, RBC, calcium, uric acid, and specific gravity of urine values, for which a weak inversely proportional relationship was noted, it can be concluded that people with the ‘Vegetables and fruits’ DP achieved optimal values of these biomarkers. In the broader literature, DPs characterized by a high intake of fruits and vegetables have also emerged in various populations [17,79,80,81,82]. The ‘Vegetables/Fruits’ DP identified in 41,351 African women aged 21–54 was associated with lower weight gain [14].

The ‘Meat and flour’ DP, which occurred more frequently in men, consisted of white meat and fried meat or flour dishes eaten several times a week. The presented DP and blood test results showed a weak, directly proportional relationship. A high, adequate supply of white meat and a moderate frequency of consumption of fried and floury foods were associated with normative HGB and RBC values, as well as higher LYMPH values. Athletes with the ‘Meat-and-flour’ DP had higher concentrations of ferritin, as well as uric acid, which can be explained by the presence of purine compounds in white meat (poultry). As in the ‘High-fat’ DP, the higher SG in urine tests, characteristic of the ‘Meat and flour’ DP, may have been related to dehydration in the athletes’ bodies. We revealed that fast food, i.e., French fries, pizza, hamburgers, etc., were consumed occasionally (~1–3 times a month) by Polish athletes, but significantly more among men than women.

The ‘Low-fat’ DP correlated weakly and directly with the indices of nutritional status assessed in this study. For athletes presenting this DP, higher ferritin levels and normal RBC levels were significant, while higher BMI and WHR values were associated with higher frequency of consumption, particularly of red meat. Achieving higher scores in the DPs for red meat, fish, lard, and fast food, despite the occasional appearance of high-fat products in the diet, was linearly associated with higher concentrations of inorganic phosphorus, vitamin B12, urea (average compound), ALT, and GGTP. Of note, elevated cobalamin concentrations were shown in 13% (*n* = 30) and inorganic phosphorus in 12% (*n* = 26) of all athletes. A stronger correlation was observed between the DP and urea levels, above-normal levels of which were recorded in 7% (*n* = 16) of athletes, including higher prevalence among men than women. Higher values of HCT and SG, similarly to the ‘High-fat’ DP, may indicate inadequate hydration of the body of athletes who presented the indicated DP. Lower %FM and normal serum total protein concentrations were significant for the ‘Low-fat’ DP. Among foods with high nutritional value, fish was consumed the least frequently. Similarly, nearly half of athletes training in martial arts reached for fish 1–2 times a week [61]. Different observations were made among Greek athletes [57]; Belgian sprinters, for whom meats and sausages (girls 19 ± 15 g/day; boys 20 ± 18 g/day) were the most popular bread garnishes [59]; and 22 Spanish athletes training taekwondo, judo, and boxing (red meat and derivatives intake exceeded the recommendations) [75]. According to Gacek and Frączek (2016) [60], fish was included by athletes several times a month, whilst Coutinho et al. (2016) [49] demonstrated that up to 90% of athletes ate fish once or less often a week.

More typical for women, the ‘Dairy’ DP was characterized by greater consumption of fermented dairy drinks, cottage cheese, and milk. As reported by Frączek (2013) [17], dairy products were consumed daily by professional athletes, and less than 75% of rowers consumed milk and milk products in at least two meals per day [50]. High consumption rates of milk and milk products were also observed in 39% of martial arts athletes [61] and 55.5% of Iranian athletes (4–7 times a week) [21], as well as among Polish female soccer players [60] and Spanish female sports students [83]. In a study on 73 Canadian athletes, up to 82% of the sample consumed milk below individual recommendations [84], which paralleled work on Canadian women [85]. Low milk supply affected 38% of Brazilian athletes training team games (basketball, handball, volleyball, soccer) [19]. Only one weak, inverse relationship was found between the described DP and serum creatinine levels, which simultaneously increased with lower frequency of consumption of milk and milk products, potentially indicating dehydration. With regard to athletes, an elevated creatinine concentration is consistent with dehydration [86]. Elevated serum creatinine levels were reported in 14% (*n* = 32) of subjects.

The ‘Juices’ DP clustered fruit juices drunk with moderate frequency, i.e., once a week on average, and vegetable and vegetable–fruit juices consumed occasionally (1–3 times a month). A weak positive correlation was found between this DP and blood biochemical indicators, such as serum magnesium, iron, and urea concentrations. At the same time, athletes who drank vegetable, fruit, and fruit–vegetable juices with similar frequency had a lower BMI, and biochemical blood tests showed lower/normal serum concentrations of chloride, total protein, creatinine, and uric acid.

One of two DPs considered unhealthy (‘High-fat’ and ‘Sweets and beverages’) showed an association with anthropometric indices. The ‘High-fat’ DP, more characteristic of men, positively correlated with the slenderness index and negatively with BMI. A potentially positive effect on nutritional status was shown for the ‘Vegetables and fruits’ DP (normal values of the AMC and BMI, uric acid concentration, and proper hydration), ‘Low-fat’ (normal %FM), ‘Dairy’ (decrease in serum creatinine), ‘Juices’ (lower BMI and decreased values of creatinine, total protein, chloride, and uric acid). Potentially, the presentation of these DPs may be related to maintenance of proper nutritional status.

The DPs identified in this study offer valuable guidance for sports dietitians, coaches, and athletes in formulating tailored nutritional strategies that align with the specific physiological demands and training modalities of various athletic disciplines. For instance, the identification of ‘High-fat’ and ‘Sweets and beverages’ patterns, predominantly observed among male athletes, underscores the need for targeted nutritional counseling aimed at moderating the intake of saturated fats and added sugars to mitigate potential adverse health effects. Conversely, the promotion of dietary patterns characterized by higher consumption of ‘Vegetables and fruits’, ‘Low-fat’, and ‘Dairy’ products may support optimal metabolic function, hydration status, and recovery, particularly among female athletes. These findings advocate for a nuanced, individualized approach to nutrition planning that integrates both macronutrient composition and hydration management, as evidenced by the associations observed between specific dietary patterns and biochemical markers of hydration. Furthermore, the results highlight the importance of incorporating sport-specific nutritional recommendations within dietary guidelines to enhance athletic performance and health outcomes.

### 4.2. Strengths and Limitations

The present study offers several notable strengths that enhance the robustness and relevance of its findings. First, the comprehensive dietary assessment allowed for the identification of eight distinct dietary patterns (DPs) among elite Polish athletes, capturing a nuanced spectrum of eating behaviors relevant to this specific population. The use of factor analysis techniques facilitated a rigorous, data-driven approach to uncovering complex interrelationships among food groups, enabling a more precise characterization of habitual dietary intake beyond single nutrients or foods. Second, the integration of biochemical markers and anthropometric indices alongside dietary data provided a multidimensional perspective on the nutritional status of athletes, allowing for the exploration of meaningful associations between dietary patterns and objective health indicators. This holistic approach strengthens the validity of the conclusions drawn regarding the potential impact of identified DPs on hydration status, metabolic markers, and body composition, which is rarely addressed in similar studies. Third, the study sample included elite-level athletes, enhancing the applicability of the findings for sports nutrition professionals aiming to optimize dietary strategies tailored to the specific physiological and metabolic demands of competitive sports. The differentiation of dietary patterns by sex and their relation to biomarkers further contributes to the understanding of gender-specific nutritional needs and risks within athletic populations. Finally, by situating the findings within the broader context of international research, the study highlights both commonalities and unique dietary trends among Polish athletes, providing valuable comparative insights that may inform culturally sensitive nutritional guidance and future research directions.

There are several study limitations. First, dietary assessment methods can represent measurement error due to the unreliability of survey participants’ responses. Consequently, we acknowledge that the food and frequency data reported in the present study may be lower or higher than that actually consumed. In addition, we acknowledge inherent limitations associated with the use of food frequency questionnaires (FFQs), including potential recall bias and limited sensitivity to capture intra-individual dietary variability. These methodological constraints may influence the accuracy and representativeness of the dietary patterns identified.

Second, it was difficult to standardize blood test results due to the characteristics of the study population (reference ranges defining normal/abnormal results are different for women and men, as well as age). In assessing the relationship between DPs and biochemical indicators, we were interested in whether they were normative or not. Therefore, it was best to assign ranks so that statistical analyzes would be performed. However, it should be noted that transforming continuous biochemical data into ranks may reduce statistical sensitivity, potentially impacting the interpretability of the results. This transformation entails a loss of detailed numerical information, which may limit the ability to detect subtle differences or correlations between variables. Additionally, rank-based analysis preserves only the order of values without reflecting the magnitude of differences between them, which could oversimplify the actual relationships present in the biochemical data. These limitations were taken into account when interpreting the findings and highlight the need for cautious conclusions regarding the associations observed.

One important limitation of the present study is the uneven representation of individual sports disciplines within the sample, which restricts the generalizability of the findings. Given the heterogeneity of physiological demands across sport types (e.g., endurance, strength, or technical disciplines), it is likely that nutritional requirements and dietary behaviors vary accordingly. However, due to sample size limitations, we did not stratify participants by sport category in the statistical analysis. As such, the study should be considered exploratory in nature, aiming to identify broad dietary patterns rather than to draw definitive conclusions applicable across all athletic populations. Future research with larger and more balanced samples is warranted to enable sport-specific analyses and to refine dietary recommendations tailored to the unique demands of different athletic disciplines.

## 5. Conclusions

In this study, eight distinct dietary patterns (DPs) were identified among Polish elite athletes, two of which (‘High-fat’ and ‘Sweets and beverages’) were considered unhealthy due to excessive intake of processed meats, fats, and sugary products. Gender differences were evident: male athletes more frequently followed high-fat, meat- and flour-based diets, whereas female athletes adhered more to vegetable- and dairy-rich patterns. Markers such as elevated serum albumin, creatinine, and urine specific gravity suggest that certain DPs—particularly those low in water-rich foods—may be linked to inadequate hydration. The dietary patterns identified in this study are original and specific to the population of Polish elite athletes, differing in structure and composition from those commonly reported in the general population or other athletic cohorts. These findings may support the development of tailored nutritional guidelines for athletes, with practical applications for coaches, sports dietitians, and health professionals working in elite sports.

## Figures and Tables

**Table 1:** Characteristics of selected somatic traits, anthropometric indices, and nutritional status indicators.

Anthropometric Parameters	Total (*n* = 226)		Women (*n* = 87)		Men (*n* = 139)		*p* Values
	x^−^	SD	x^−^	SD	x^−^	SD	
**Age (years)**	22.8	5.5	21.7	4.6	23.5	5.9	0.330
**Body height (cm)**	177.5	9.2	**170.8**	7.8	**181.7**	7.2	**<0.001**
**Body weight (kg)**	69.9	12.2	**61.6**	**8.3**	**75.0**	11.3	**<0.001**
**Circumference (cm)**							
**Arm at rest**	29.9	3.3	**28.2**	2.6	**30.6**	3.3	**<0.001**
**Waist**	78.4	6.9	**73.2**	5.3	**80.7**	6.2	**<0.001**
**Hip**	91.6	6.0	91.3	5.7	91.7	6.2	0.706
**Skin-fat folds (mm)**							
**Over the triceps arm muscle**	9.6	4.9	**12.8**	5.1	**8.2**	4.1	**<0.001**
**Over the biceps muscle of the arm**	5.4	2.9	**7.0**	3.7	**4.8**	2.3	**<0.001**
**Under the shoulder blade**	10.7	4.7	10.4	3.9	10.8	5.0	0.870
**Over the iliac crest**	11.4	6.4	11.7	5.3	11.3	6.9	0.115
**Sum of four skin-fat folds**	37.2	16.3	**41.9**	15.2	**35.2**	16.3	**<0.001**
**Anthropometric indices**							
**BMI (kg/m^2^)**	22.1	2.5	**21.1**	2.1	**22.7**	2.6	**<0.001**
**WHR**	0.9	0.1	**0.8**	0.1	**0.9**	0.0	**<0.001**
**Slenderness**	43.3	1.5	43.4	1.5	43.2	1.6	0.838
**AMC (cm)**	26.9	3.3	**24.2**	2.3	**28.0**	3.0	**<0.001**
**%FM**	17.3	6.3	**23.5**	5.2	**14.6**	4.5	**<0.001**

BMI—body mass index (kg/m^2^), WHR—waist–hip ratio, AMC—arm muscle circumference, %FM—percentage of fat mass, x^−^—averages, SD—standard deviation, *p*—significance level of the chi-square test.

**Table 2:** Peripheral blood composition, biochemical blood tests, and urine test in a group of athletes—distribution of results.

Indices	%									
	Total			Women			Men			
	1	2	3	1	2	3	1	2	3	*p* Values
HGB (g/dL/g/L/mmol/L)	0.6	98.8	0.6	0.0	100.0	0.0	0.9	98.2	0.9	0.261
HCT (%)	0.6	97.6	1.8	0.0	98.1	1.8	0.9	97.3	1.8	0.427
RBC (mln/μL/T/L)	3.0	94.6	2.4	0.0	96.3	3.7	4.5	93.7	1.8	0.289
MCV (fl)	1.8	91.0	7.2	**3.7**	**83.3**	**13.0**	**0.9**	**94.6**	**4.5**	**0.005**
MCH (pg/fmol)	0.0	97.6	2.4	0.0	98.1	1.9	0.0	97.3	2.7	0.792
MCHC (g/dL/g/L/mmol/L)	4.8	94.5	0.6	3.8	94.3	1.9	5.4	94.6	0.0	0.061
RDW-CV (%)	4.4	91.2	4.4	**6.0**	**84.0**	**10.0**	**3.6**	**94.5**	**1.8**	**0.005**
PLT (tys/μL/G/L)	3.0	95.8	1.2	1.8	98.1	0.0	3.6	94.6	1.8	0.208
WBC (tys/μL/G/L)	9.0	88.5	2.4	5.6	90.7	3.7	10.7	87.5	1.8	0.109
NEUT (tys/μL/G/L)	15.1	84.8	0.0	20.4	79.6	0.0	12.6	87.4	0.0	0.122
LYMPH (tys/μL/G/L)	0.0	90.8	9.1	0.0	90.7	9.3	0.0	90.9	9.1	0.968
MONO (tys/μL/G/L)	0.0	98.2	1.8	0.0	100.0	0.0	0.0	97.3	2.7	0.110
EOS (tys/μL/G/L)	0.6	97.5	1.8	**1.9**	**98.1**	**0.0**	**0.0**	**97.3**	**2.7**	**0.017**
BASO (tys/μL/G/L)	12.8	85.4	1.8	**9.3**	**90.7**	**0.0**	**14.5**	**82.7**	**2.7**	**0.044**
Serum sodium (mmol/L)	0.0	98.7	1.3	0.0	100.0	0.0	0.0	98.1	1.9	0.168
Serum potassium (mmol/L)	0.6	96.7	2.6	0.0	98.0	2.0	0.9	96.2	2.9	0.402
Serum chloride (mmol/L/mg/dL)	0.9	97.3	1.8	0.0	97.4	2.6	1.3	97.3	1.3	0.224
Total serum calcium (mmol/L/mg/dL)	0.7	86.6	12.7	2.5	87.5	10.0	0.0	86.3	13.7	0.055
Serum inorganic phosphorus (mmol/L/mg/dL)	0.9	87.4	11.7	0.0	83.8	16.2	1.3	89.2	9.5	0.063
Serum magnesium (mmol/L/mg/dL)	1.3	94.7	4.0	4.3	93.6	2.1	0.0	95.2	4.8	0.065
Serum iron (mmol/L/μmol/L/mol/L/U/L/μg/dL)	7.3	87.1	5.6	8.9	88.9	2.2	6.3	86.1	7.6	0.062
Serum ferritin (mmol/L/ng/mL/μg/L)	4.0	89.7	6.3	**8.3**	**81.2**	**10.4**	**1.3**	**94.9**	**3.8**	**0.001**
Serum vitamin B12 (pg/mL/μg/L)	1.6	85.4	13.0	2.2	82.2	15.6	1.3	87.2	11.5	0.278
Total serum protein (g/L/g/dL)	1.3	95.3	3.4	**2.3**	**97.7**	**0.0**	**0.9**	**94.3**	**4.7**	**0.019**
Serum albumin (g/L/g/dL)	0.0	73.9	26.1	**0.0**	**89.2**	**10.8**	**0.0**	**68.3**	**31.7**	**<0.001**
Serum urea (μmol/L/mg/dL)	3.3	89.5	7.19	**8.5**	**91.5**	**0.0**	**0.9**	**88.7**	**10.4**	**<0.001**
Serum creatinine (μmol/L/μg/dL)	2.6	83.8	13.7	**0.0**	**80.5**	**19.5**	**3.9**	**85.5**	**10.5**	**0.013**
Serum uric acid (mmol/L/mg/dL)	2.9	91.4	5.7	4.2	91.7	4.2	2.2	91.3	6.5	0.246
Blood glucose (mmol/L/mg/dL)	0.0	92.8	7.2	**0.0**	**100.0**	**0.0**	**0.0**	**89.6**	**10.4**	**0.002**
Triglycerides level (mmol/L/mg/dL)	0.0	96.6	3.4	**0.0**	**100.0**	**0.00**	**0.0**	**95.1**	**4.8**	**0.034**
Total cholesterol level (mmol/L/mg/dL)	5.3	74.7	20.0	4.4	77.8	17.8	5.7	73.3	21.0	0.417
LDL cholesterol level (mmol/L/mg/dL)	0.0	87.8	12.2	**0.0**	**97.7**	**2.3**	**0.0**	**83.6**	**16.3**	**0.001**
HDL cholesterol level (mmol/L/mg/dL)	3.4	95.2	1.4	2.3	95.3	2.3	3.9	95.1	1.0	0.256
Total serum bilirubin (μmol/L/mg/dL)	0.0	87.4	12.6	**0.0**	**95.8**	**4.2**	**0.0**	**83.5**	**16.5**	**0.007**
AST (U/L)	0.0	82.7	17.3	**0.0**	**71.1**	**28.9**	**0.0**	**87.6**	**12.4**	**0.002**
ALT (U/L)	0.0	95.3	4.7	0.0	95.6	4.4	0.0	95.2	4.8	0.882
GGTP (Ul/L)	3.4	95.3	1.35	2.3	95.5	2.3	3.8	95.2	1.0	0.256
Urine color	0.0	59.4	40.6	0.0	65.2	34.9	0.0	56.5	43.5	0.195
Urine pH	0.0	97.1	2.9	0.0	97.8	2.2	0.0	96.8	3.2	0.792
Urine specific gravity (g/mL/kg/L/mg/dL)	8.0	84.0	8.0	11.1	84.4	4.4	6.2	83.8	10.0	0.057
Protein in urine	0.0	98.5	1.4	0.0	100.0	0.0	0.0	97.8	2.2	0.168
Glucose in urine	0.0	99.3	0.7	0.0	100.0	0.0	0.0	98.9	1.1	0.261
Ketone bodies in urine	0.0	91.2	8.8	0.0	87.0	13.0	0.0	93.4	6.6	0.112
Bilirubin in urine	0.0	100.0	0.0	0.0	100.0	0.0	0.0	100.0	0.0	1.000
Urobilinogen in urine	0.0	100.0	0.0	0.0	100.0	0.0	0.0	100.0	0.0	1.000
Nitrites in urine	0.0	100.0	0.0	0.0	100.0	0.0	0.0	100.0	0.0	1.000

Key: 1—below the norm, 2—within the norm, 3—above the norm. *p*—significance level of the Chi-square test.

**Table 3:** Factor loading matrix for the eight DPs identified from the food frequency questionnaire (FFQ).

Dietary Patterns (*n* = 226).	Food and Food Products	Factor Loadings	Variance Explained (%)
**High-fat**	Light bread	0.71	8.7
	Potatoes	0.64	
	Cold cuts, wieners, sausages	0.63	
	Cheese, processed cheese, moldy cheese	0.61	
	Butter	0.49	
**Sweets** **and beverages**	Sweetened carbonated and non-carbonated drinks	0.67	6.8
	Sweets	0.58	
	Alcoholic beverages	0.55	
	Energy drinks	0.55	
	Powdered or ready-made soups	0.54	
**Potentially rational**	Eggs	0.69	6.2
	Legume seeds	0.68	
	White rice, pasta, small groats	0.57	
	Canned, marinated, or pickled vegetables	0.44	
	Buckwheat groats, flakes, whole-grain pasta	0.42	
**Vegetables** **and fruits**	Vegetables	0.71	5.7
	Fruits	0.69	
**Meat and flour**	White meat	0.74	5.6
	Fried foods	0.61	
**Low-fat**	Red meat	0.71	5.4
	Fish	0.59	
	Lard	0.58	
	Fast food	0.42	
**Dairy**	Fermented milk drinks	0.79	5.3
	Cottage cheese	0.69	
	Milk	0.54	
**Juices**	Vegetable juices, vegetable and fruit juices	0.75	4.7
	Fruit juices	0.63	

Method of extracting factors—main components; rotation method—Varimax with Kaiser normalization; factor loadings with absolute values ≥0.40.

**Table 4:** Characteristics of the derived DPs in a group of athletes.

DPs.	Positive Relationship	Negative Relationship
High-fat	slenderness index ^1^, HCT ^1^, RBC ^1^, serum iron ^1^, serum urea concentration ^1^, TG ^1^, SG ^1^	BMI ^1^, serum uric acid ^1^, AST ^1^
Sweets and beverages	PLT ^1^, WBC ^1^, NEUT ^1^, serum albumin ^1^, serum uric acid ^1^, GGTP ^1^	AST ^1^
Potentially rational	BMI ^1^, BASO ^1^	slenderness index ^1^, serum chloride ^1^, ketone bodies ^1^
Vegetables and fruits	-	BMI ^1^, AMC ^1^, HGB ^1^, HCT ^1^, RBC ^1^, serum calcium ^1^, serum uric acid ^1^, SG ^1^
Meat and flour	HGB ^1^, RBC ^1^, LYMPH ^1^, MONO ^1^, serum ferritin ^1^, serum uric acid ^1^, SG ^1^	%FM ^1^
Low-fat	BMI ^1^, WHR ^1^, HCT ^1^, RBC ^1^, serum inorganic phosphorus ^1^, serum ferritin ^1^, serum vit. B12 ^1^, ALT ^1^, GGTP ^1^, serum urea ^2^, SG ^1^	%FM ^1^, total serum protein ^1^
Dairy	-	serum creatinine ^1^
Juices	serum magnesium ^1^, serum iron ^1^, serum urea ^1^	BMI ^1^, serum chlorine ^1^, total serum protein ^1^, serum creatinine ^1^, serum uric acid ^1^

Correlation strength |r|: ^1^—weak, ^2^—average. The strength of the correlation for most of the analyzed variables was shown to be ‘weak.’ HCT—hematocrit, HGB—hemoglobin, RBCs—red blood cells, WBCs—white blood cells, PLT—platelet count, TG—triglycerides level, SG—urine specific gravity, BMI—body mass index, AMC—arm muscle circumference, %FM—percentage of fat mass, AST—aspartate transaminase, GGTP—γ-glutamyl transferase, ALT—alanine aminotransferase, LYMPH—lymphocytes level, MONO—monocytes level.

**Table 5:** Spearman’s rho correlations between anthropometric indices, body fat percentage, selected peripheral blood biochemical indices and DPs based on FFQ.

DPs								
Nutritional Status Indicators	High-Fat	Sweets and Beverages	Potentially Rational	Vegetables and Fruits	Meat and Flour	Low-Fat	Dairy	Juices
**BMI [kg/m^2^]**	−0.17 *	0.06	0.17 **	−0.14 *	0.05	0.15 *	−0.06	−0.18 **
**WHR**	0.02	0.05	0.04	−0.04	0.07	0.22**	−0.07	−0.07
**Slenderness**	0.24 **	0.00	−0.16 *	0.06	0.10	−0.11	0.05	0.12
**AMC [cm]**	−0.02	0.02	0.15	−0.25 **	0.13	0.10	−0.09	−0.13
**%FM**	−0.13	0.01	−0.02	0.13	−0.16 *	−0.16 *	0.13	−0.09
**HGB (g/dL/g/L/mmol/L)**	0.10	0.10	−0.05	−0.26 **	0.16 *	0.14	−0.11	−0.06
**HCT (%)**	0.16 *	0.07	0.02	−0.27 **	0.18 *	0.18 *	−0.13	−0.02
**RBC (mln/μL/T/L)**	0.16 *	0.07	0.02	−0.27 **	0.18 *	0.18 *	−0.13	−0.02
**MCV (fl)**	0.04	−0.12	0.03	−0.04	−0.04	0.05	0.11	0.09
**MCH (pg/fmol)**	0.01	−0.08	−0.03	−0.05	−0.02	0.03	0.00	0.10
**MCHC (g/dL/g/L/mmol/L)**	−0.01	0.10	−0.10	0.01	0.09	−0.13	−0.05	−0.04
**RDW-CV (%)**	0.13	−0.15	0.04	0.02	−0.12	−0.02	0.09	0.03
**PLT (tys/μL/G/L)**	−0.05	0.18 *	0.06	0.04	−0.11	0.14	−0.03	−0.05
**Triglycerides level (mmol/L/mg/dL)**	0.13	0.17 *	0.05	−0.01	0.14	0.03	0.00	−0.12
**Total cholesterol level (mmol/L/mg/dL)**	0.09	0.18 *	0.05	0.00	0.02	0.00	0.03	−0.09
**LDL cholesterol level (mmol/L/mg/dL)**	0.13	0.08	−0.06	−0.06	0.17 *	0.07	−0.01	−0.09
**HDL cholesterol level (mmol/L/mg/dL)**	0.00	0.11	0.13	−0.09	0.21 **	−0.09	−0.01	−0.07
**EOS (tys/μL/G/L)**	−0.01	0.07	0.01	−0.11	0.10	0.13	−0.07	−0.07
**BASO (tys/μL/G/L)**	−0.01	0.07	0.18 *	0.01	0.12	−0.05	0.00	−0.10
**Serum sodium (mmol/L)**	−0.04	0.09	−0.011	0.03	0.12	−0.05	−0.15	−0.15
**Serum potassium (mmol/L)**	−0.15	−0.11	0.06	0.07	0.07	0.15	−0.05	−0.03
**Serum chloride (mmol/L/mg/dL)**	0.15	−0.05	−0.25 **	−0.04	−0.01	−0.04	−0.08	−0.28 **
**Total serum calcium (mmol/L/mg/dL)**	0.08	−0.01	0.03	−0.19 *	−0.13	0.14	0.06	0.10
**Serum inorganic phosphorus (mmol/L/mg/dL)**	0.10	−0.10	−0.02	−0.09	−0.04	0.21 *	0.08	0.15
**Serum magnesium (mmol/L/mg/dL)**	−0.12	−0.02	−0.10	0.01	−0.13	0.14	0.11	0.20 *
**Serum iron (mmol/L/** **μ** **mol/L/mol/L/U/L/** **μ** **g/dL)**	0.22 *	−0.05	−0.07	−0.05	−0.06	0.13	0.13	0.22 *
**Serum ferritin (mmol/L/ng/mL/μg/L)**	0.16	0.04	−0.03	−0.16	0.18 *	0.23 **	−0.11	−0.07
**Serum vitamin B12 (pg/mL/** **μ** **g/L)**	−0.08	−0.08	0.07	−0.06	0.01	0.25 **	0.09	0.10
**Total serum protein (g/L/g/dL)**	−0.05	0.14	0.05	−0.03	0.05	−0.20 *	0.05	−0.21 *
**Serum albumin (g/L/g/dL)**	−0.15	0.17 *	0.02	−0.08	0.00	–0.08	0.06	−0.16
**Serum urea (μmol/L/mg/dL)**	0.19 *	−0.12	0.04	−0.08	0.07	0.32 **	0.11	0.17 *
**Serum creatinine (μmol/L/μg/dL)**	−0.09	0.14	0.00	−0.10	0.12	−0.01	−0.20 *	−0.20 *
**Serum uric acid (mmol/L/mg/dL)**	−0.17 *	0.19 *	−0.02	−0.18 *	0.25 **	−0.03	−0.15	−0.29 **
**Blood glucose (mmol/L/mg/dL)**	0.10	−0.08	−0.14	0.00	0.08	0.07	−0.08	−0.05
**Triglycerides level (mmol/L/mg/dL)**	0.18 *	−0.08	0.01	−0.08	−0.05	0.15	0.05	0.07
**Total cholesterol level (mmol/L/mg/dL)**	0.05	−0.15	0.06	0.02	−0.12	0.10	0.00	0.09
**LDL cholesterol level (mmol/L/mg/dL)**	0.07	−0.15	0.05	−0.04	−0.10	0.08	0.01	0.10
**HDL cholesterol level (mmol/L/mg/dL)**	0.12	−0.11	0.15	0.14	−0.13	0.10	0.01	0.16
**AST (U/L)**	−0.23 **	−0.17 *	0.11	−0.04	0.08	0.02	0.12	0.02
**ALT (U/L)**	−0.08	−0.09	0.10	−0.07	0.12	0.19 *	0.00	−0.06
**GGTP (Ul/L)**	0.09	0.21 *	0.02	−0.06	0.08	0.24 **	−0.02	−0.09
**Urine color**	0.13	−0.01	−0.11	−0.14	0.04	0.07	0.03	0.09
**Urine pH**	−0.11	−0.05	−0.03	0.05	0.10	−0.12	−0.13	0.05
**Urine specific gravity (g/mL/kg/L/mg/dL)**	0.17 *	0.16	−0.04	−0.23 **	0.22 *	0.18 *	–0.10	−0.02
**Protein in urine**	−0.01	−0.09	0.02	0.00	0.02	0.06	0.04	0.05
**Glucose in urine**	−0.04	0.14	0.08	−0.08	0.11	−0.11	−0.14	0.12
**Ketone bodies in urine**	−0.01	0.11	−0.17 *	−0.03	0.13	−0.07	0.08	−0.01

Key: correlations *: *p* < 0.05, **: *p* < 0.01.

## Data Availability

The final data generated during this study are included in this article and its Supplementary Information Files. In addition, primary datasets are available from the corresponding author upon reasonable request.

## Supplementary Materials

The following supporting information can be downloaded at https://www.mdpi.com/article/10.3390/nu17162685/s1: Table S1: Distribution of the studied athletes (%) by BMI, WHR, slenderness index, AMC, and %FM classification; Table S2: Average frequency of consumption of food products in a group of athletes; Table S3: Intergroup differences in DPs based on FFQ.

## Abbreviations

The following abbreviations are used in this manuscript:

%FM	Fat mass percentage
ALT	Alanine aminotransferase
AMC	Arm muscle circumference
AST	Aspartate transaminase
BASOs	Basophils
BMI	Body mass index
DPs	Dietary patterns
EOSs	Eosinophils
FA	Factor analysis
FFQ	Food frequency questionnaire
GGTP	γ-glutamyl transferase
HCT	Hematocrit
HDL-c	High-density lipoprotein cholesterol
HGB	Hemoglobin
KMO	Kaiser–Meyer–Olkin
LDL-c	Low-density lipoprotein cholesterol
LYMPHs	Lymphocytes
MAC	Midarm circumference
MCH	Mean corpuscular hemoglobin
MCHC	Mean corpuscular hemoglobin concentration
MCV	Mean corpuscular volume
MONOs	Monocytes
NEUT s	Neutrophils
PCA	Principal component analysis
PLT	Platelet count
RBCs	Red blood cells
RDW-CV	Red blood cell distribution width
SG	Specific gravity
TGs	Triglycerides
WBCs	White blood cells
WHR	Waist–hip ratio
