# Supplementary material for: Dietary Patterns and Nutritional Status of Polish Elite Athletes
Source: Nutrients. 2025 Aug 19;17(16):2685. doi: 10.3390/nu17162685 (PMC12389033; doi:10.3390/nu17162685)
Supplement: Supplementary file 1 [file nutrients-17-02685-s001.zip › nutrients-3792221-supplementary.pdf]

Table S1. Distribution of the studied athletes (%) by BMI, WHR, slenderness index, AMC and %FM classification.

BMI classification (kg/m²)	Total *n*=226	Women *n*=87	Men *n*=139
		*chi*²(3) = 17.96; $p < 0.001$	
3rd degree of leanness (<16.0)	0.0	0.0	0.0
2nd degree of leanness (16.0–16.9)	0.0	0.0	0.0
1st degree of leanness (17.0–18.4)	4.9	11.5	0.7
Norm (18.5–24.9)	84.1	83.9	84.2
Overweight (25.0–29.9)	9.7	4.6	12.9
1st degree of obesity (30.0–34.9)	1.3	0.0	2.2
2nd degree of obesity (35.0–39.9)	0.0	0.0	0.0
3rd degree of obesity (>40.0)	0.0	0.0	0.0
**WHR classification**		*chi*²(3) = 25.32; $p < 0.001$	
Great (W: <0.75, M: <0.85)	15.1	10.0	17.4
Good (W: 0.75–0.79, M: 0.85–0.89)	43.4	30.0	49.5
Medium (W: 0.80–0.85, M: 0.90–0.95)	36.5	48.0	31.2
High (W: 0.86–0.90, M: 0.96–1.00)	3.8	8.0	1.8
Extremely high (W: >0.90, M: >1.00)	1.3	4.0	0.0
**Slenderness index classification**		*chi*²(2) = 18.82; $p < 0.001$	
Endomorphy (W: <40.7, M: <39.5)	3.5	4.6	2.9
Mesomorphy (W: 40.7–44.2, M: 39.5–43.2)	54.0	70.1	43.9
Leanness (Ectomorphy) (W: >44.2, M: >43.2)	42.5	25.3	53.2
**AMC classification**		*chi*²(2) = 8.21; $p = 0.017$	
Above standard	76.4	62.0	82.6

Good (W: 23.2–20.9, M: 25.3–22.8)	20.0	32.0	14.8
Slight malnutrition (W: 20.8–18.6, M: 22.7–20.2)	3.6	6.0	2.6
Moderate malnutrition (W: 18.5–16.2, M: 20.1–17.7)	0.0	0.0	0.0
Severe malnutrition (W: <16.2, M: <17.7)	0.0	0.0	0.0
**%FM by Siri equations**		$chi^2(4) = 3.72$; $p = 0.445$	
Low (W<18, M: <8)	7.3	12.0	5.2
Optimal (W: 18-20, M: 8-10)	9.1	12.0	7.8
Correct (W: 20-22, M: 10-12)	13.3	10.0	14.8
High (W: 22-24, M: 12-14)	18.2	16.0	19.1
Very high (W: >24, M: >14)	52.1	50.0	53.0

Key: BMI – body mass index (kg/m²), WHR – waist-hip ratio, AMC – arm muscle circumference, %FM – percentage of fat mass, W – women, M – men

Table S2. Average frequency of consumption of food products in a group of athletes.

**Food product and/or dish**	**Total**		**M**		**SD**		***P Values***
	**M**	**SD**	**Women**	**Men**	**Women**	**Men**	
**Light bread**	4.50	1.43	**4.15**	**4.71**	1.52	1.33	0.005
**Wholemeal bread**	3.84	1.33	3.94	3.78	1.38	1.31	0.411
**White rice, pasta, small groats**	4.07	1.06	**3.70**	**4.30**	1.18	0.90	<0.001
**Buckwheat groats, flakes, whole grain pasta**	3.93	1.20	4.11	3.82	1.22	1.18	0.061
**Fast food**	2.22	0.75	**2.02**	**2.34**	0.70	0.76	<0.001
**Fried foods**	3.78	1.16	3.68	3.85	1.20	1.14	0.172
**Butter**	3.95	1.71	3.90	3.98	1.79	1.67	0.802
**Lard**	1.42	0.88	1.16	1.59	0.53	1.01	<0.001
**Oils, margarines, mixes**	2.10	1.65	**1.80**	**2.29**	1.46	1.73	0.041
**Milk**	4.02	1.56	4.08	3.99	1.60	1.54	0.401
**Fermented milk drinks**	3.85	1.21	3.99	3.76	1.23	1.18	0.081

**Cottage cheese**	3.45	1.27	**3.66**	**3.32**	1.27	1.26	0.049
**Yellow cheeses, processed cheeses, moldy cheeses**	3.83	1.26	**3.59**	**3.99**	1.28	1.23	0.018
**Cold cuts, sausages, wieners**	4.06	1.36	**3.55**	**4.38**	1.40	1.24	<0.001
**Red meat**	3.23	1.12	**2.79**	**3.50**	1.12	1.03	<0.001
**White meat**	4.17	0.98	4.03	4.26	1.06	0.93	0.313
**Fish**	2.83	0.93	2.80	2.84	1.01	0.89	0.962
**Eggs**	3.97	0.90	4.06	3.91	0.78	0.97	0.415
**Legume seeds**	2.77	1.06	2.79	2.75	1.18	0.99	0.842
**Potatoes**	3.65	1.02	3.51	3.74	1.13	0.94	0.081
**Fruits**	5.08	0.95	**5.32**	**4.94**	0.93	0.93	0.001
**Vegetables**	5.01	1.01	**5.29**	**4.83**	0.99	0.98	<0.001
**Sweets**	3.92	1.34	**4.14**	**3.78**	1.37	1.30	0.043
**Powdered or ready-made soups**	1.37	0.79	**1.21**	**1.47**	0.57	0.88	0.010
**Canned meats**	1.44	0.74	**1.23**	**1.58**	0.52	0.82	0.001
**Canned, marinated and pickled vegetables**	2.25	1.16	2.36	2.18	1.19	1.14	0.267
**Fruit juices**	3.50	1.35	3.34	3.60	1.44	1.29	0.179
**Vegetable, vegetable and fruit juices**	2.81	1.45	3.01	2.69	1.49	1.42	0.114
**Sweetened hot drinks**	5.08	1.23	**5.31**	**4.93**	1.04	1.31	0.028
**Sweetened carbonated and non-carbonated beverages**	2.52	1.22	**2.18**	**2.73**	1.31	1.12	<0.001
**Energy drinks**	1.66	0.96	**1.48**	**1.78**	0.89	0.99	0.008
**Water**	5.72	0.83	**5.84**	**5.65**	0.68	0.91	0.016
**Alcoholic beverages**	2.08	0.96	**1.92**	**2.19**	0.94	0.95	0.015

Key: M – mean, SD – standard deviation, *p* – significance level of the Mann–Whitney U test

Table S3. Intergroup differences in DPs based on FFQ.

**DPs**	**M**		**SD**		***Student t-test***	***p Values***
	**Women**	**Men**	**Women**	**Men**		
**High-fat**	**-0.27**	**0.17**	**1.02**	**0.95**	-3.24	0.001
**Sweets and beverages**	-0.03	0.02	0.95	1.04	-0.39	0.700
**Potentially rational**	0.00	0.00	1.04	0.98	-0.04	0.968
**Vegetables and fruits**	**0.28**	**-0.18**	**0.92**	**1.01**	3.44	0.001
**Meat and flour**	**-0.21**	**0.13**	**1.01**	**0.97**	-2.48	0.014
**Low-fat**	**-0.36**	**0.23**	**0.90**	**1.00**	-4.48	<0.001
**Dairy**	**0.18**	**-0.11**	**0.99**	**1.00**	2.14	0.033
**Juices**	0.07	-0.04	1.05	0.97	0.85	0.394

Key: M – mean, SD – standard deviation, *p* – significance level of the Student's t-test
